# Genome Sequence of *Synechococcus* sp. Strain LA31, Isolated from a Temperate Estuary

**DOI:** 10.1128/mra.00775-21

**Published:** 2022-03-09

**Authors:** Joshua D. Kling, Eric A. Webb, David A. Hutchins

**Affiliations:** a Department of Biological Sciences, University of Southern California, Los Angeles, California, USA; Montana State University

## Abstract

Cluster 5 *Synechococcus* species are widely acknowledged for their broad distribution and biogeochemical importance. In particular, subcluster 5.2 strains inhabit freshwater, estuarine, and marine environments but are understudied, compared to other subclusters. Here, we present the genome for *Synechococcus* sp. strain LA31, a strain that was recently isolated from Narragansett Bay, Rhode Island, USA.

## ANNOUNCEMENT

Unicellular picocyanobacteria from the genus *Synechococcus* are found throughout aquatic environments (1–3) and play a key role in global carbon cycling (4). However, the biodiversity and biogeochemical contributions of nonmarine *Synechococcus* strains are only beginning to be understood (3). Here, we present the genome of *Synechococcus* sp. strain LA31, which was recently isolated from a temperate estuary.

LA31 was isolated from the Narragansett Bay Time Series in Rhode Island, USA (latitude, 41.47; longitude, −71.40). To enrich for phytoplankton, surface water (22°C; salinity, 28.84 psu) was spiked with nutrients comparable to F/40 medium for 10 days (5) and given 150 μmol photons/m^−2^ · s^−1^ of light (12-h light/12-h dark cycle). Single cells were sorted into 96-well plates using an Influx flow cytometer (BD, San Jose, CA, USA) and were maintained under the same conditions. Wells showing growth after 2 weeks were streaked onto F/2 agar plates for isolation, and one colony was transferred to F/2 medium (5). DNA was extracted using a series of freeze-thaw cycles in N_2_, proteinase K incubations, and final extraction with the Qiagen DNeasy PowerBiofilm kit (Hilden, Germany), as described previously (6). MR DNA (Shallowater, TX, USA) performed library preparation (SMRTbell Express template preparation kit v2.0; Pacific Biosciences), sequencing (Sequel system), read quality control, and assembly (Hierarchical Genome Assembly Process [HGAP] in single-molecule real-time [SMRT] Analysis v9.0). Assembly completeness and contamination were measured with CheckM v1.1.3 (7). Gene calling and annotation were done with Prodigal v2.6.3 (8) and KofamScan v1.3.0 (9). antiSMASH v6.0 (10) was used for detection of secondary metabolite genes, tRNAscan-SE v2.0 for detection of tRNA sequences (11), and Barrnap v0.9 for detection of rRNA sequences (12). A phylogenomic tree of all unicellular picocyanobacterial assemblies available in the NCBI RefSeq database (13) was constructed using GToTree v1.5.51 with the included cyanobacterial marker gene set (14–18). Default parameters were used for all software unless otherwise noted.

**FIG 1:**
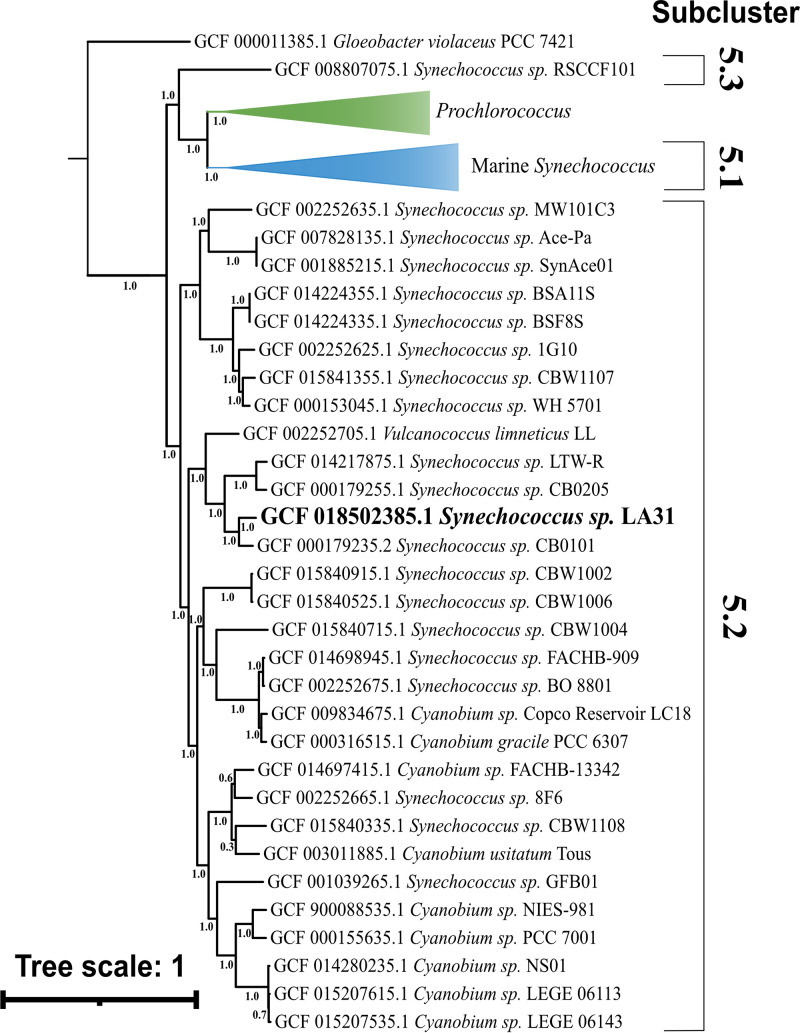
Maximum likelihood tree created with 251 concatenated amino acid sequences found in all currently available cluster 5 *Synechococcus*, *Cyanobium*, *Vulcanococcus*, and *Prochlorococcus* genomes. The distribution of the three subclusters within cluster 5 is indicated with brackets on the right side. Strain LA31 (presented here) is shown in bold. *Gloeobacter violaceus* PCC 7421 is included as an outgroup. Node numbers represent local support values derived from 1,000 resamplings as part of FastTree. Genbank accession numbers are listed for each genome.

A total of 1,209,177 reads (average length, 3,532.17 bp) were assembled into a single contig 2,752,051 bp in length (GC content, 63%; completeness, 99.46%; contamination, 0.54%). A total of 2,965 coding regions were detected (1,501 annotated), with 47 tRNAs and three 5S, 16S, and 23S rRNA genes. A phylogenetic tree of 251 conserved proteins from 219 genomes placed LA31 in *Synechococcus* subcluster 5.2 (Fig. 1), branching with both brackish (CB0101 [19]) and freshwater (*Vulcanococcus limneticus* LL [20]) isolates.

Nitrogen transporters for nitrate/nitrite (*NRT* [*n* = 1]), ammonium (*AMT* [*n* = 2]), urea (*urtABCDE* and *Dur3*), and 15 amino acids (21) were detected in LA31. A purine/hypoxanthine transporter was also detected; such transporters are upregulated in eukaryotes under N deprivation but have not been reported in cyanobacteria (22). High-affinity phosphate uptake systems (*pstABC* and *pstS* [*n* = 3]) and phosphonate transporters (*phnD* and *phnE*) were detected for P, and Fe transporters were detected for both ferrous (*feoAB*) and ferric (*fbpAB* or *idiAB*) (23) Fe. Four toxin-antitoxin gene pairs were detected, similar to closely related CB0101 (24). Thirteen secondary metabolite coding regions were identified for ribosomally synthesized and posttranslationally modified peptides (RiPPs) (*n* = 10), terpene synthesis (*n* = 2), and hierridin B (*n* = 1), a potential antimalarial compound (25).

### Data availability.

This assembly is available under GenBank accession number CP075523 and RefSeq assembly accession number GCF_018502385.1. The version described is the first version. Reads are available under SRA accession number SRR14511408.
